# Reflexive attention can be driven by monocular cues without conscious access to eye of origin information

**DOI:** 10.1007/s00221-026-07269-y

**Published:** 2026-03-09

**Authors:** Luis J. Fuentes, Fernando Valle-Inclán

**Affiliations:** 1 Departamento de Psicología Básica y Metodología, Universidad de Murcia, Murcia, Spain; 2 Departamento de Psicología, Universidad de La Coruña, La Coruña, Spain

**Keywords:** Monocular cueing, Reflexive attention, Eye-of-origin information, Dichoptic presentation, Unconscious attention

## Abstract

This study examined whether monocular cues can facilitate reflexive attention in the absence of conscious access to the origin of the cue. Across three experiments, participants viewed brief, task-irrelevant, non-predictive cues, followed by targets presented through a mirror stereoscope. The cue and target were delivered to the same eye (valid trials) or the opposite eye (invalid trials). In Experiment 3 only, the cue was presented binocularly in 25% of trials (the neutral condition). Participants could not reliably discriminate which eye received the stimuli and responded solely to target detection. Reaction times were reliably faster in valid trials than in invalid or neutral trials at a cue-target stimulus-onset asynchrony (SOA) of 100 ms. No cueing effects were observed at shorter or longer SOAs, and no evidence of inhibition of return emerged. These findings suggest that early attentional facilitation can be triggered by eye-specific signals, even when there is no conscious awareness of the cued eye. By demonstrating a temporally selective, eye-based facilitation effect, the present results extend previous work on unconscious attentional modulation. They also suggest that early sensory representations can bias reflexive attentional orienting independently of awareness.

## Introduction

Visual awareness and attention have long been treated as closely related processes, both in classical theories of perception and in early experimental paradigms, where attentional selection was often assumed to imply conscious access (e.g., Posner 1980; Treisman and Gelade 1980). However, converging evidence accumulated over the past two decades has challenged this view, demonstrating that attention and awareness can be functionally and neurally dissociated. In particular, attentional selection can operate on sensory signals that fail to reach conscious awareness, suggesting a fundamental distinction between attention as a selection or prioritization mechanism and consciousness as a subjective experience (Chelazzi et al. 2018; Jiang et al. 2015; Lamme 2003, 2004; Maier et al. 2022).

A particularly informative demonstration of this dissociation comes from studies employing monocular stimulation. In these paradigms, the same visual stimuli are presented to one eye at a time, so that the eye of origin is the only feature that differentiates the input. Although observers are usually unable to consciously report the eye of origin, this information is preserved at the earliest stages of visual processing. Monocular stimulation tasks typically exploit this property by associating task-relevant signals, or attentional priorities, with one eye while participants perform an explicit visual task that does not require access to the eye-of-origin information. Importantly, these eye-specific associations can influence attentional selection and modulate behavioral performance despite remaining outside of conscious awareness, providing compelling evidence for eye-based attentional guidance in the absence of visual awareness (Zhaoping 2008). For instance, eye-of-origin cues have been shown to affect the perception of partially occluded objects (Rensink and Enns 1998), perceptual dominance in binocular rivalry (Ooi and He 1999, 2020), forced-choice judgments above chance even in the absence of subjective awareness (Blake and Cormack 1979), and orientation discrimination of Gabor targets (Kim and Chong 2022). These findings indicate that monocular information can be processed and exploited by the visual system without becoming consciously accessible.

Further support for unconscious attentional processing comes from studies of perceptual learning under dichoptic or interocular suppression conditions. Rosenthal et al. (2010) showed that participants could learn higher-order visuospatial sequences presented monocularly, even when the eye-of-origin and spatial layout of the sequence remained masked from conscious awareness via mirror stereoscopy. Similarly, Verleger et al. (2004) showed that attentional effects can be shaped by perceptual parameters such as stimulus visibility and spatial layout independently of awareness. Taken together, these findings suggest that early, eye-specific representations (preserved in structures such as the lateral geniculated nucleus (LGN) and primary visual cortex (V1), can guide behavior even when their defining properties remain inaccessible to consciousness (Dougherty et al. 2019a, b; see Maier et al. 2022 for a review).

Despite this growing body of evidence, a key theoretical issue remains unresolved: to what extent can transient, reflexive attentional orienting be driven by monocular signals in the absence of awareness, and what is the temporal profile of such effects? While several studies have demonstrated that eye-of-origin information can influence performance, the tasks they employed differed substantially in terms of their demands, timing and the extent to which attentional dynamics were characterised. For instance, Zhaoping (2008) demonstrated that ocular singletons could guide attention in visual search tasks even when the eye-of-origin was not perceived. However, these effects were measured using brief or simultaneous stimulus presentations and did not involve the systematic manipulation of cue–target onset asynchrony (SOA). In contrast, Self and Roelfsema ( 2010) used a Posner-type cueing paradigm with explicit SOAs and found that there was facilitation for monocularly cued targets only at very short intervals (50 ms), with no effects observed at longer SOAs. This finding suggests a highly transient influence of monocular cues, potentially linked to early competition mechanisms, but leaves open the question of whether such effects generalize across a broader temporal window or engage later inhibitory dynamics.

Importantly, existing studies have not systematically examined whether monocular cueing effects adhere to the standard temporal pattern of exogenous attention: an initial facilitatory phase followed by inhibition of return (IOR). The absence of IOR in previous monocular cueing studies may be due to the selection of SOAs that did not sufficiently sample the timeframe in which inhibitory effects usually appear (approximately 300 ms in detection tasks and up to 700 ms or more in discrimination tasks; Klein 2000; Lupiáñez et al. 1997). As a result, it is unclear whether monocular cues can engage the full temporal dynamics of reflexive attention or whether their influence is restricted to an early, short-lived facilitatory phase.

In the present study, we address this gap by adopting a classical peripheral cueing paradigm involving non-predictive exogenous cues, alongside a systematic manipulation of cue-target SOAs spanning both facilitatory and inhibitory ranges. Using a mirror stereoscope, we presented brief monocular cues, followed by targets at various SOAs, ensuring that participants were unaware of which eye was stimulated. Participants were instructed to respond to targets as quickly as possible. We tested whether responses would be faster on valid trials (when the cue and target were presented to the same eye) than on invalid trials (when the cue and target were presented to different eyes) at short SOAs, and whether this pattern would reverse at longer SOAs, which would be consistent with IOR.

By characterizing the temporal limits of eye-specific cueing effects, the present study aims to clarify the conditions under which reflexive attention can be driven by monocular signals in the absence of awareness.

## Method

### Participants

Forty-eight participants were recruited through the university-wide mailing list. Experiment 1 tested 16 participants (15 females), aged 18–22 years (M = 19.2, SD = 1.3). Experiment 2 tested 12 participants (all female), aged 19–25 years (M = 21.1, SD = 1.8). Experiment 3 tested 20 participants (all female), aged 19–23 years (M = 20.6, SD = 1.4). All participants reported normal or corrected-to-normal vision and signed a written consent at the beginning of the experiment. Participants received course credits for their participation. The study was approved by the Ethics Committee of the University of Murcia and was performed in accordance with the ethical standards laid down in the 1964 Declaration of Helsinki.

### Stimuli and materials

Stimuli were presented on a 60 Hz VGA monitor (Sony Multiscan 17 SE, resolution 1024 × 768 pixels) in a dimly lit testing room. Participants viewed the stimuli through a mirror stereoscope for dichoptic presentation, 40 cm away from the screen, and rested their chins on a chin rest. Two alignment squares ensured binocular fusion. Cue stimuli were white squares, and the target consisted of the letter “H” in white. Responses were recorded through the computer keyboard.

### Procedure

Before the experimental session, participants aligned the two squares through the mirror stereoscope. Participants had to move the two squares closer or further apart with the arrow keys on the keyboard until the merging of the two squares was perceived as a single square presented in the center, thus ensuring binocular fusion. Then, they completed a block of 60 trials in which only the target letter was presented, randomly to either the left square (left eye) or right square (right eye). They were instructed to indicate which eye they believed had been stimulated by pressing the “C” key for the left eye and the “M” key for the right eye. This screening block served to exclude participants who achieved a percentage of correct responses exceeding 55%. Using a cut-off of 55% in a 60-trial two-alternative forced choice (2AFC) task is consistent with current recommendations for defining chance-level performance in low-trial psychophysical assessments.

Recent methodological work emphasizes that small deviations from 50% in short binary tasks are to be expected under binomial noise and should not be interpreted as genuine sensitivity (e.g., García-Pérez and Alcalá-Quintana 2012). In contrast, performance levels below the exclusion cut-off were treated as reflecting guessing rather than partial awareness, and no feedback was provided that could have allowed participants to exploit correct guesses during the task. For 60 trials, these bounds extend to approximately 62–63%, meaning that observed accuracies within this range are consistent with null sensitivity. By adopting a more conservative threshold of 55%, we ensured that minor, noise-driven fluctuations around chance performance were not misclassified as perceptual discrimination. This criterion, therefore, provides a robust basis for concluding that participants lacked reliable conscious access to eye-of-origin information. As none of the participants exceeded this awareness criterion, none were excluded from the experiments.

**Figure 1:**
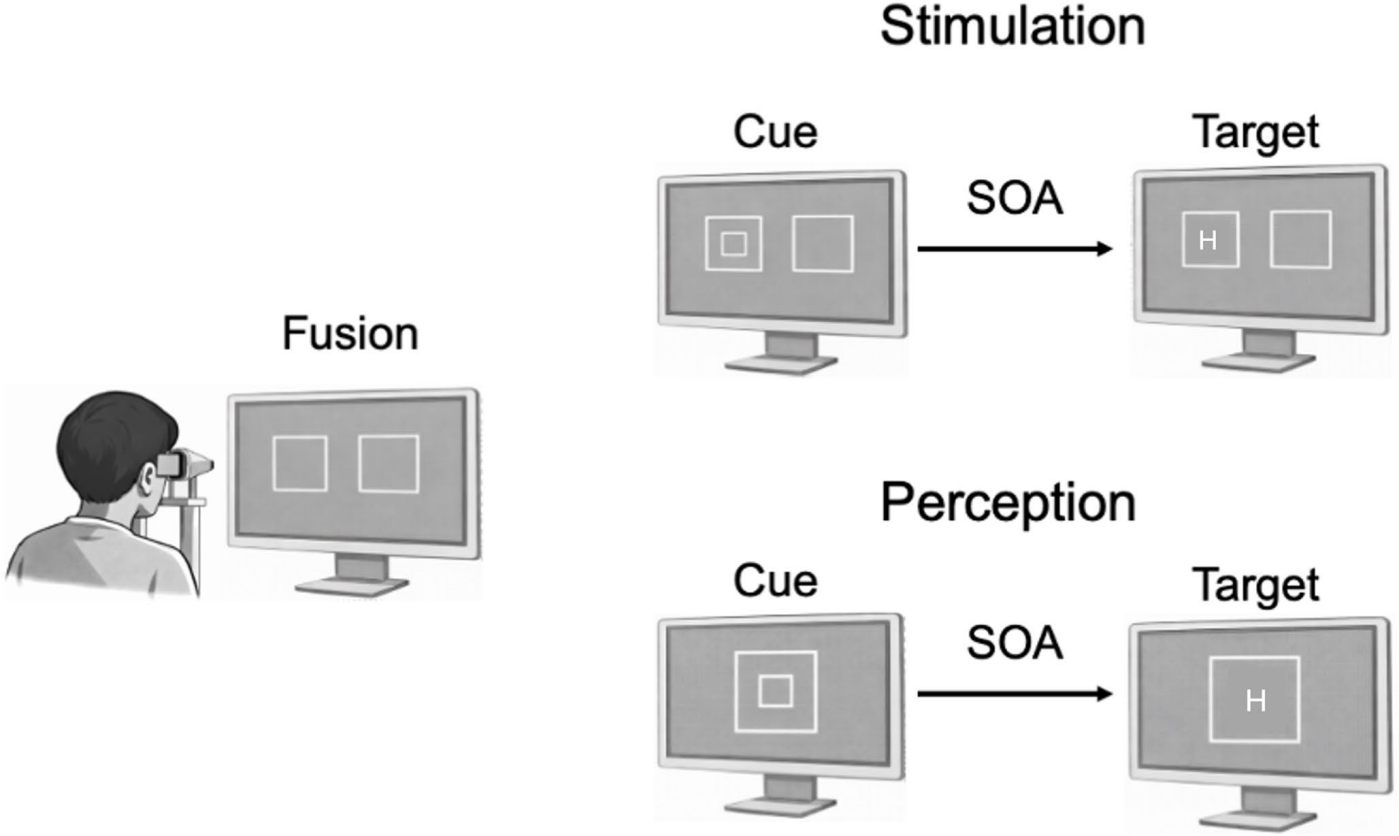
Fig. 1 Participants performed the experiment by looking through a stereoscope. During the fusion phase, they moved the two squares closer together or further apart until they saw a single square in the centre of the screen. The stimuli presented in the left square stimulated only the left eye, and those presented in the right square stimulated only the right eye. ‘Stimulation’ refers to how the stimuli were presented in each trial. In valid trials, the cue (small square) and the target (H) were presented in the same square, thereby stimulating the same eye (left or right). In invalid trials, the cue was presented in one of the side squares and the target in the other, thus stimulating different eyes. Only Experiment 3 included neutral trials, in which the cue was presented bilaterally in both squares. ‘Perception’ refers to how the participant perceives both the cue and the target, i.e., as being in the centre of the screen, regardless of which eye was stimulated

For the experimental session, the two squares previously fused for each participant were permanently presented to each eye during a block of trials. Seen through the stereoscope, the fused image was a gray square of 6°, with a line width of 0.5°, presented in front of the participant, in a black background. The cue was a white square of 2.2°, with a line width of 0.5°, concentric with the fusion square. After a variable delay (see below), the target letter was monocularly presented and perceived as presented in the center of the fused square (see “Perception” in Fig. 1).

On cue-present trials, the monocular cue appeared randomly in one of the side squares for 60 ms (Experiment 1) or 28 ms (Experiments 2 and 3). Following an SOA of 0, 50, 100, 200, 400 or 800 ms (Experiment 1), or 50, 100 or 200 ms (Experiments 2 and 3), the target letter was presented monocularly for 150 ms in one of the side squares (see “Stimulation” in Fig. 1). In Experiment 1, at the 0 ms SOA, the cue and target onsets were simultaneous. In valid trials, the target “H” was embedded within the small square cue and was therefore presented in the same side square, stimulating the same eye. In invalid trials, the cue and target were presented simultaneously within opposite side squares, stimulating different eyes. Although the cue and the target overlapped temporally at 0 ms, they were perceptually distinct and therefore easily discriminable. Participants pressed the “H” key as quickly as possible upon detection.

According to “Stimulation” (Fig. 1), a trial was considered valid if the cue and target were presented to the same side square (i.e., the same eye) and invalid if they were presented to opposite side squares (i.e., different eyes). In Experiments 1 and 2, there were only valid and invalid trials, and the cue was spatially non-informative regarding the target location (50% valid trials). In Experiment 3, 25% of trials also included binocular cues, i.e., the cue was presented in both side squares, stimulating both eyes (the neutral condition). The luminance of the binocular cues was adjusted so that they were perceived as monocular cues. Importantly, throughout the study, the cue validity variable was defined in terms of correspondence of the eye of origin rather than spatial location as commonly defined in classical Posner-type cueing paradigms.

The three experiments were designed as sequential steps to test the robustness and interpretation of monocular cueing effects. Experiment 1 explored a wide range of SOAs to characterize the temporal profile of the effect. Experiment 2 served as a replication that focused on the SOA range in which facilitation was observed, using shorter cue durations. Experiment 3 replicated Experiment 2 but introduced a binocular-neutral cue condition to separate the facilitatory benefits from the inhibitory costs. The instructions for Experiment 3 emphasized speeded responding to increase sensitivity to early facilitation effects.

Each participant completed a practice block of 20 trials, followed by 8 experimental blocks of 64 trials in Experiments 1 and 2, and 6 blocks of 80 trials in Experiment 3. Trials were randomized within blocks; 25% of trials were catch trials (no target) to monitor false alarms. The inter-trial interval was 1000 ms. Participants were encouraged to maintain central fixation and stable stereoscopic alignment throughout.

### Data analysis

Reaction times (RTs) were recorded for correct target detections. The first trial of each block and trials with RTs < 150 ms or > 600 ms were excluded (< 5% of trials). Responses faster than 150 ms were excluded as anticipatory, as such latencies are unlikely to reflect stimulus-driven perceptual or attentional processing. Responses slower than 600 ms were excluded as they are more susceptible to attentional lapses, delayed decision processes, or variability in motor execution. Using this temporal window is consistent with standard practice in exogenous attentional cueing and RT paradigms, where intermediate response time (RT) ranges are considered to be the most sensitive to facilitatory and inhibitory attentional effects. Importantly, the same RT window was applied uniformly across all SOAs, cueing conditions, and experiments. This ensured that any observed effects could not be attributed to differential response filtering. False alarms on catch trials were also excluded.

Mean RTs for valid and invalid (Experiments 1 and 2), and valid, invalid and neutral (only in Experiment 3) conditions were computed as a function of SOA. Variance analyses (ANOVAs) were conducted separately for each experiment, with factors Cue Validity (2 levels: valid, invalid in Experiments 1–2; or 3 levels: valid, invalid, neutral, in Experiment 3), SOA, and their interactions. Greenhouse-Geisser corrections were applied when Mauchly’s sphericity test was violated.

In addition to univariate repeated-measures ANOVAs, we report multivariate tests of within-participant effects using Pillai’s trace as a robustness check. This test reflects a multivariate approach to repeated-measures analysis. In this approach, the levels of the within-participant factors are treated as a vector of repeated observations of the same dependent variable and do not assume sphericity (Tabachnick and Fidell 2019). Results are primarily interpreted based on univariate analyses with Greenhouse-Geisser correction, and multivariate statistics are reported for completeness.

We adopted a statistical significance level of α = 0.05 for all statistical analyses. In addition, we present effect size values for our contrasts using partial eta squared (*h*_p_^2^). Pairwise comparisons used paired-samples *t*-tests with Holm-Bonferroni correction.

## Results

**Figure 2:**
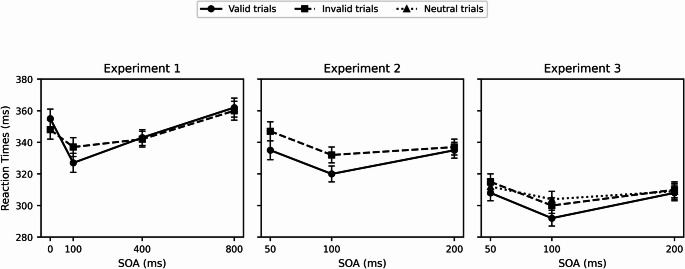
Fig. 2 Mean reaction time and standard error bars as a function of SOA and Cue Validity for Experiment 1 (left), Experiment 2 (middle), and Experiment 3 (right). Circles represent valid trials, squares represent invalid trials, and triangles represent neutral trials (Experiment 3 only)

**Experiment 1**. Mean RTs and standard errors are displayed in Fig. 2 (left). There was a significant main effect of SOA, *F*(3,45) = 6.64, *p* < .001, *h*_p_^2^ = 0.31 (Pillais’s trace test of significance *F*(3,13) = 15.92, *p* < .0001), indicating faster responses at intermediate SOAs (100 ms: M = 332 ms; 400 ms: M = 343 ms) than at 0 ms (M = 353 ms) and 800 ms (M = 361 ms). Cue Validity also had a significant main effect, *F*(1,15) = 6.11, *p* = .025, *h*_p_^2^ = 0.29, with faster responses on valid trials (M = 339 ms) than invalid trials (M = 346 ms). The SOA × Cue Validity interaction approached statistical significance, *F*(3,45) = 2.63, *p* = .062, *h*_p_^2^ = 0.15, but it reached statistical significance with the multivariate test (Pillais’s trace test of significance, *F*(3,13) = 4.02, *p* < .03). Post hoc comparisons showed that the cue validity effect was 10 ms at 100 ms SOA, *F*(1,15) = 14.99, *p* < .002, and absent in the other three SOAs. False alarms on catch trials were rare (M = 1.61%), indicating high task compliance. No evidence of IOR was observed at longer SOAs (400 ms and 800 ms).

**Experiment 2**. Mean RTs and standard errors are displayed in Fig. 2 (middle). There was a significant main effect of SOA, *F*(2,22) = 7.89, *p* < .003, *h*_p_^2^ = 0.42 (Pillais’s trace test of significance, *F*(2,10) = 17.22, *p* < .001). RTs were faster with 100 ms SOA (M = 325 ms) than with both 50 ms SOA (M = 341 ms) and 200 ms SOA (M = 337 ms). Cue Validity was also significant, *F*(1,11) = 8.10, *p* = .016, *h*_p_^2^ = 0.42. The interaction between SOA and Cue Validity was significant, *F*(2,22) = 4.08, *p* = .03, *h*_p_^2^ = 0.27 (Pillais’s trace test of significance, *F*(2,10) = 5.36, *p* < .03). Post hoc comparisons revealed just a significant cue validity effect at 100 ms (13 ms), *F*(1,11) = 17.39, *p* = .002. The effect decreased at 50 ms SOA (10 ms), where it did not reach statistical significance, *F*(1,11) = 3.54, *p* = .089; and it was completely absent at 200 ms SOA (− 1 ms). False alarms occurred on 5.56% of catch trials, slightly higher than in Experiment 1, but still within acceptable limits.

**Experiment 3**. Mean RTs and standard errors are displayed in Fig. 2 (right). There was a significant main effect of SOA, *F*(2,38) = 11.85, *p* < .001, *h*_p_^2^ = 0.38 (Pillais’s trace test of significance, *F*(2,18) = 22.95, *p* < .0001). RTs were faster with 100 ms SOA (M = 298 ms) than with both 50 ms SOA (M = 312 ms) and 200 ms SOA (M = 310 ms). The Cue Validity main effect was also significant, *F*(2,38) = 6.54, *p* < .004, *h*_p_^2^ = 0.26 (Pillais’s trace test of significance, *F*(2,18) = 5.01, *p* < .02). Valid trials were faster than invalid and neutral trials. However, these main effects were modulated by the significant SOA × Cue Validity interaction, *F*(4,76) = 2.83, *p* < .03, *h*_p_^2^ = 0.13 (Pillais’s trace test of significance, *F*(4,16) = 3.21, *p* < .04). Post hoc comparisons showed that at 50 ms SOA there were not significant differences between valid trials (M = 308 ms) and both neutral (M = 314 ms) nor invalid trials (M = 315 ms), *F*(2,38) = 3.19, *p* < .06 (Pillais’s trace test of significance, *F*(2,18) = 3.1, *p* < .07). At 100 ms SOA, RTs were significantly faster on valid trials (M = 292 ms) than on neutral (M = 304 ms) and invalid trials (M = 299 ms), *F*(2,38) = 8.96, *p* < .001 (Pillais’s trace test of significance, *F*(2,18) = 9.65, *p* < .001. No significant differences were found at 200 ms SOA. Critically, neutral and invalid trials did not differ significantly at any SOA, suggesting that cueing effects were due to benefits (facilitation) rather than costs (inhibition). Catch trial responses were higher in this experiment (M = 8.19%), likely due to the stronger emphasis on speeded responses in instructions.

## Discussion

Although attention and awareness have historically been treated as closely related processes, contemporary theoretical and empirical work has emphasized their partial dissociation, showing that attentional guidance can occur without awareness (e.g., Chelazzi et al. 2018; Jiang et al. 2015). Within this framework, the present study provides behavioral evidence that reflexive attention can be guided by monocular cues in the absence of conscious access to eye-of-origin information.

Across three experiments, we observed faster responses to targets shown to the same eye as a preceding, non-predictive cue, with the cueing effects limited to a short time frame (100 ms SOA). Participants were unaware of which eye was stimulated, and the inclusion of a binocular-neutral condition in Experiment 3 confirmed that facilitation rather than suppression explains the observed effects. These results support the idea that attentional selection can operate on implicit, early-stage visual representations, and add to the evidence that spatial attention effects can occur when cues are presented below the threshold of conscious perception (Krishnakumar et al. 2024; McCormick 1997; see Schoeberl et al. 2015 for similar findings in attention capture tasks).

A notable aspect of the results is the distinction between the initial facilitatory effects and the absence of inhibition of return (IOR). Inhibition of return represents an extraordinary adaptive function of the orientation system that encourages the organism to explore new locations by both preventing attention from returning to already attended locations (Klein 2000; Posner and Cohen 1984) and affecting the processing of stimuli falling in those locations (Fuentes et al. 1999; for a review, see Fuentes et al. 2012). A preference for searching novel locations may be crucial for organisms to survive, and it might explain why it develops so early in infancy (Clohessy et al. 1991). Our results suggest that conscious access to spatially definable cue information may be necessary for IOR to emerge, in line with previous studies that showed a lack of inhibitory processing in the absence of awareness (see Fuentes and Humphreys 1996, for similar evidence using a negative priming task with a neglect patient). By contrast, the facilitation effect at short intervals does develop without awareness of the cue and stimulus spatial locations.

At first glance, the facilitation effect without the inhibitory one (IOR) may seem unremarkable, indicating that monocular cues are effective at directing attention towards task-relevant stimuli but not at directing it away at later time points. However, this dissociation is theoretically informative. It implies that the facilitatory and inhibitory components of exogenous attention differ in their representational format and computational requirements, despite both being considered hallmarks of reflexive orienting. Early facilitation can be interpreted as a transient gain modulation that enhances the processing of recently cued inputs. Such a mechanism can plausibly operate on local, implicit representations, including eye-of-origin signals preserved at early stages of the visual system. In contrast, IOR may require additional operations, including tagging previously attended locations, maintaining selection history over time and updating attentional priority maps. These operations likely depend on spatially definable coordinates that can be referenced and compared across events. However, when the cue is defined solely by the eye of origin and remains inaccessible to awareness, such a spatial reference frame may be unavailable, preventing inhibitory processing from emerging, despite early facilitation remaining intact. This interpretation is consistent with broader accounts of unconscious attention, which emphasize that selection and prioritization can occur without awareness. However, processes requiring temporal integration or contextual updating are more constrained (Chelazzi et al. 2018).

More broadly, the present dissociation affects how exogenous orienting is conceptualized. Exogenous attention is often described as exhibiting a characteristic temporal profile, with early facilitation followed by IOR. IOR has sometimes been considered a defining feature of stimulus-driven orienting. However, the present findings challenge this characterization, showing that robust short-latency facilitation can occur without a subsequent IOR phase. As mentioned before, this is particularly true when selection is driven by implicit, eye-specific signals that do not provide an accessible spatial reference for inhibitory tagging. This pattern is consistent with previous studies indicating that IOR can be separated from other forms of spatial orienting, depending on task demands and representational constraints (Chica et al. 2006, 2014). Taken together, these results support a more nuanced, component-based view, in which exogenous attention is not treated as a single, complete mechanism but rather as a set of partially dissociable processes. Some of these processes (e.g., early gain facilitation) can be triggered by implicit unconscious sensory cues, while others (e.g., IOR) require additional computations, such as spatially explicit tagging and priority-map updating over time.

However, before drawing firm conclusions, alternative explanations must be considered. The results do not align with an inhibition-based account, whereby validity effects stem from the suppression of the non-cued eye. Such an account predicts that RTs will increase in invalid trials as the SOA increases, reflecting the gradual development of inhibition. Instead, RTs decreased globally at the 100 ms SOA for both valid and invalid trials, while a relative advantage for valid trials remained. Moreover, Experiment 3 showed that invalid and neutral trials produced comparable RTs, whereas only valid trials exhibited facilitation. This pattern is inconsistent with an inhibitory mechanism and instead supports a facilitation-based account.

A further alternative is that the cue transiently biases interocular balance, such that targets presented to the same eye benefit from a short-lived dominance state (e.g., Wolfe 1984). We agree that flash-triggered interocular dynamics are a principled concern in dichoptic paradigms. However, Wolfe’s single-flash reversal paradigm differs from the present design in several respects, including sustained monocular stimulation before probing, the presence of a dark interstimulus interval, and the use of simultaneous binocular test flashes to infer dominance via interocular competition. By contrast, our cues were brief (28–60 ms), embedded within a stable fusion frame, and targets were presented monocularly. These differences limit a direct one-to-one mapping from Wolfe’s psychometric time course to the present RT validity effect.

Empirically, the eye-specific benefit was most reliable at the 100 ms SOA across three experiments and absent at 200 ms and longer SOAs. While the lack of statistical significance at 50 ms cannot by itself exclude interocular accounts (given comparable effect sizes and higher variability), Experiment 3 provides an additional constraint: neutral (binocular cues) and invalid trials did not differ, whereas valid trials were faster than both. This pattern indicates a benefit specific to same-eye correspondence without measurable costs for the opposite eye. Accordingly, although we cannot rule out that transient interocular dynamics contribute to early eye-specific differences, the present data are more parsimoniously described as a short-latency benefit linked to cue-target correspondence in the eye of origin rather than as a generalized suppression of the non-cued eye. Overall, the timing and selectivity of the observed effects are consistent with the idea of rapid, eye-specific gain modulation. However, they do not provide clear support for a purely rivalry-driven or suppression-based account. Nevertheless, we acknowledge that ocular dominance dynamics may contribute to early eye-specific differences in dichoptic stimulation paradigms.

A final alternative interpretation is that the facilitation observed at 100 ms reflects non-specific alerting rather than selective attentional modulation. Although a global reduction in RTs was indeed observed at this SOA, alerting alone cannot account for the validity effects. Crucially, valid and invalid trials involved identical monocular cues and therefore conveyed the same temporal warning signal. Under a purely alerting account, RTs should be equally reduced in both conditions, yielding no cue-target correspondence effects. Instead, a reliable valid-invalid difference was observed at 100 ms and replicated across the three experiments. Furthermore, the introduction of a binocular neutral condition revealed a pure benefit pattern (valid < neutral ≈ invalid), demonstrating facilitation without accompanying costs. These findings suggest that alerting may provide a permissive temporal window within which selective, eye-specific facilitation can operate, rather than serving as a sufficient explanation.

The dissociation between attention and awareness observed here aligns with theoretical models positing distinct functional roles for these processes (Maier and Tsuchiya 2021; Tsuchiya and Koch 2016). While attention enhances sensory processing and modulates prioritization, it does not necessarily entail conscious access. In the present study, attentional orienting was driven by monocular features that participants could not reliably discriminate, reinforcing the view that unconscious attention can influence perceptual selection (Self and Roelfsema 2010; Zhaoping 2008).

Our findings also resonate with work by Rosenthal et al. (2010), who showed that participants could learn higher-order visuospatial sequences under stereoscopic conditions that masked spatial information from awareness. As in the present study, learning occurred without conscious monitoring of target positions or spatially aligned motor responses. Together, these findings underscore the capacity of the visual system to process and exploit monocular or retinotopic information outside the scope of introspection.

From a neural perspective, the present results do not imply that attentional computations are confined to early visual structures such as the lateral geniculate nucleus or the primary visual cortex. These regions are highlighted because they preserve eye-of-origin information and provide a plausible basis for the early, eye-specific gain modulation reflected in the short-latency facilitation observed here, and because they have been shown to support attentional modulation without accompanying awareness (Zhaoping 2008, 2019). However, attentional orienting is known to rely on distributed networks, including the superior colliculus and frontoparietal systems, which compute attentional priority and salience (Chica et al. 2011; Veale et al. 2017). Importantly, invoking early visual structures does not entail a strict mapping between specific brain regions and conscious versus unconscious processing, as frontoparietal networks can also mediate attentional effects without explicit awareness (Jiang et al. 2015).

The present findings contribute to growing evidence that early sensory mechanisms can guide attentional selection in the absence of awareness. Within multistage models of consciousness, such as Global Neuronal Workspace theory (Dehaene and Naccache 2001; Mashour et al. 2020), the observed effects likely reflect attentional modulation occurring prior to the amplification and integration processes required for conscious access, shaping behavior without entering subjective experience.

Several limitations should be acknowledged. First, the sample size was not determined by an a priori power analysis, which constrains the strength of population-level inferences. However, the critical pattern of results, a selective eye-specific facilitation at 100 ms accompanied by a global alerting effect, was replicated across three independent experiments, increasing confidence in the reliability of the findings. Future studies with larger samples and formal power analyses will be necessary to refine effect size estimates and assess generalizability. Second, another limitation concerns the absence of a post-experiment assessment of eye-of-origin awareness. Although participants did not exceed chance performance in the initial screening block, it remains possible that sensitivity increased during the experiment. Nonetheless, if participants had gained reliable conscious access to eye-of-origin information, one would expect stronger, more sustained, or later-emerging cueing effects, none of which were observed. Thus, while absolute unawareness cannot be claimed, the data indicate the absence of reliable conscious discrimination throughout the task.

In sum, the present findings demonstrate that monocular cues can elicit robust, short-latency attentional facilitation without giving rise to later inhibitory effects. This dissociation refines current accounts of exogenous attention by showing that its canonical components do not necessarily co-occur when selection is driven by implicit sensory signals. More broadly, the results support multistage models of attention and consciousness, in which attentional orienting comprises a sequence of partially dissociable operations, only some of which can be implemented without conscious access to spatially explicit representations. 

## Data Availability

Data are available upon request.
